# Insight of effects of air quality and sustainable aviation fuel blend on energy saving and emission reduction in airport

**DOI:** 10.1186/s40643-024-00798-w

**Published:** 2024-09-04

**Authors:** Ziyu Liu, Sha Yu, Xiaoyi Yang

**Affiliations:** 1 School of Energy and Power Engineering, Energy and Environment International Centre, Beihang University, Beijing, China; 2 Sinopec Shanghai Engineering Company Limited, Shanghai, China

**Keywords:** Air quality, PM, UHC, Airport, SAFs, Engine emission

## Abstract

Air quality in airport attracts a widespread attention due to the emission of GHGs and pollutants related with aircraft flight. Sustainable aviation fuel (SAF) has confirmed PM_2.5_ reduction due to free of aromatics and sulphur, and thus air quality improvement in airport is prospected by SAF blend. Two types of SAF were assessed the potential of energy saving and emission reduction by ZF850 jet engine. FT fuel is characterized with only paraffins without aromatics and cycloparaffins while HCHJ fuels is characterized with no aromatics. The descend of air quality and SAF blend were both investigated the effect on the engine performance and emission characteristic. The critical parameters were extracted from fuel compositions and air pollutants. Ambient air with a higher PM_2.5_ could lead to the rise of engine emission especially in UHC and PM_2.5_ despite at the low thrust setting and high thrust setting, and even couple with 3.2% rise in energy consumption and 1% reduction in combustion efficiency. CO, NO and NO_2_ in ambient air show less influence on engine performance and emission characteristic than PM_2.5_. Both types of SAF blend were observed significant reductions in PM_2.5_ and UHC. PM_2.5_ reduction obtained 37.9%—99.8% by FT blend and 0.64%-93.9% by HCHJ blend through the whole trust settings. There are almost 6.67% positive benefit in TSFC through the whole thrust setting by 7% FT blend. The effects of air quality and SAF blend on engine emission present significant changes on PM and UHC but the slight change on CO and NO_x_. By SAF blend, the energy saving and pollutant reduction obtained could be both benefit for air quality improvement in airport and further reduce engine emission as the feedback of less pollutants in ambient air.

**Graphical Abstract:**

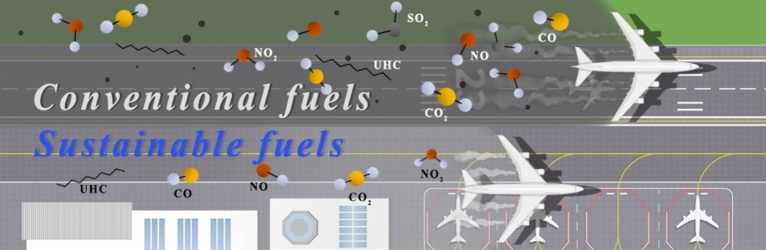

## Highlights

- Reduction of fuel consumption and engine emission by SAF blends;
- Higher PM in ambient air leads to the increase of fuel consumption and engine emission;
- Significantly reducing PM and UHC in airport by SAF blends;

## Introduction

The descend of air quality in airport attracts widespread attention due to mass centralized discharge of GHGs and pollutants with regard the issue of aircraft flight. The emissions in airport are derived from main engine and APU in aircraft, ground support equipment and vehicle (Han et al. 2022). Aircraft emission is the main discharge source in the airport, which is mainly related with LTO cycle including approach, taxi, takeoff and climb below altitude of 915 m. The total emission in a LTO cycle, could change with aircraft types and actual operation modes. Aircraft engine emits CO, SO_2_, CO_2_, H_2_O, NO_x_, unburned hydrocarbon (UHC), particulate matter (PM) and other trace compounds in combustion process, some of which as the precursor could be further transferred into secondary pollutants in the atmosphere (Stacey et al. 1994). In eight major busy airports (Turgut and Rosen 2010), significant differences were detected and concluded that every airport has a landing and take-off (LTO) cycle carried out by aircraft with different emission characteristics. According to the combustion mechanism, the current findings can be deduced that combustion productors and induced secondary productors could further enhance aircraft engine emission, and thus air quality in airport could lead to further degradation. However, from the studies assessing the contribution of airport operations on ambient air quality, the results were quite similar in PM (Stacey 2019). Several airports were discovered PM and UHC above background of city. As many studies have confirmed a strong correlation of the exposure to PM with some significant adverse human health effects, it is crucial to reduce PM emission in airport. Sustainable aviation fuels (SAFs) provide an alternative energy source with the less GHGs emission (Liu and Yang 2023a) and even bioproducts (Allen, et al. 2018).As SAFs are usually free of aromatic and sulfurs, reduction of PM emission were confirmed at engine level compared with conventional jet fuels (Liu and Yang 2023b; Liu et al. 2023a). Therefore, it is prospected to improve ambient air quality in airport by SAFs blend.

PM close to airports was confirmed to be significantly higher than location and upwind of airport, and the particle size distribution was different from traditional road traffic, which was characterized with more extreme fine particles. PM was found above background concentration in several airports such as Los Angeles international, Hartsfield‐Jackson in Atlanta, and Logan airport in Boston (Riley, et al. 2021). PM concentrations at landing approach paths could be several times higher than background concentrations (Hudda et al. 2014; Shirmohammadi et al. 2017; Ungeheuer et al. 2021; Arter and Arunachalam 2021). According to detection in Copenhagen Airport (Winther et al. 1994), emissions of NO_x_ and PM were mainly derived from main engine and APU of aircraft, and wherein main engines were responsibility for 87% NO_x_, 61% PM mass and 95% PM number as the largest source. In Tianjin airport (Ren et al. 2016), the atmospheric particle number concentration was found mainly by takeoff and landing of aircraft. At Los Angeles International Airport (Shirmohammadi et al. 2017), PM was investigated four times greater adjacent to the airport than on nearby major freeways. Moreover, PM enhancement was investigated at even several kilometers away of Boston Logan International Airport (Hudda et al. 2018). In the vicinity of Logan airport, PM concentration derived from aviation activities could get average 2 and 1.33‐fold higher at sites 4.0 and 7.3 km away from the airport (Hudda et al. 2016), and PM was detected higher in areas under landing jet trajectories (Hudda and Fruin 2016). Similarly, PM can be even increased to 3 to fivefold in transects under landing approach pathways in Los Angeles International Airport (Hsu et al. 2013; Penn et al. 2015) and Hartsfield‐Jackson International Airport (Riley et al. 2016). Ambient air can be enhanced by PM_2.5_ 8.06μg /m^3^ with PM_2.5_ 149 t/year at Beijing capital international airport (BO, X., et al. 2017). PM can penetrate deeper into the respiratory tract and into cells possibly posing some significant adverse human health effects. Therefore, it is urgent to reduce PM in airport crowded with people. PM in airport can be reduced by air purification including nanoporous membrane materials for separation (Castro-Muñoz et al. 2023; Castro-Muñoz et al. 1996; Russo et al. 2022; Castro-Muñoz and Fíla 2020; Wang et al. 2008), or from optimization of aviation activities.

Unburned hydrocarbons (UHC) are formed from the incomplete combustion of fuel in combustor of engine. UHC emitted mainly include unburned fuel compositions and oxidized or pyrolyzed combustion byproducts. Among the hydrocarbons emitted in aircraft exhaust, some species including formaldehyde, acetaldehyde, acrolein and polycyclic organic matter are considered as the hazardous air pollutants. The concentration of UHC in airport were found above background as same as PM. Ambient air can be enhanced UHC 71.89μg /m^3^ at UHC 543 t/year at Beijing capital international airport (BO, X.,, et al. 2017). The most important emission source of UHC in the airport can be attributed to aircraft emission by 67.4%, 92.2% of which was derived from aircraft taxiing (Yang et al. 2018). With respect to the effect of fuel composition on the formation of a specific species, significant reductions in both benzene and toluene were observed for the paraffinic fuels, and UHC emission was found to be sensitive to the fuel composition (Cain et al. 2013). The effect of aromatic blend to a neat FT fuel was shown that the UHC emissions increased by 13%—58% at the addition of the 20% aromatics with various molecular structures (DeWitt et al. 2008). The high molecular weight aromatic resulted in the large increase in UHC emission. For FT blend, the largest reduction was observed at 7% power for the Jet A1 with 18.5% aromatic content compared with 100% FT fuel (Lobo et al. 2011), which was consistent with the result on military engines burning FT fuels (Corporan et al. 2007). PM emission level and microphysical properties were altered with the change of fuel composition, particularly by the type and fraction of aromatic species (Schripp et al. 2022). The lower aromatic-content alternative jet fuels not only produce fewer PM emissions, and the particles were smaller (Moore et al. 2017) with different morphologies as determined by electron microscopy (Liati et al. 2019), which could even lessen their ice-forming ability. The largest reductions usually observed at idle and low power conditions (Schripp et al. 2022) and the reductions were related with a function of fuel characteristics (Corporan, et al. 2012).The relative reduction was significantly higher at lower engine power. As significant reduction in PM number emission could reduce the climate impact of contrail induced by soot (Burkhardt et al. 2018), PM reduction associated with burning SAF blend could decrease the number concentration and lifetime of contrail ice particle, and thus partially mitigate the climate impact (Kleine et al. 2018).

Most results confirmed NO_x_ and CO above background concentration in airport (Riley, et al. 2021; Hudda et al. 2020). At Beijing capital international airport (BO, X.,, et al. 2017), ambient air can be enhanced by CO 842.08 μg /m^3^ and NO_x_ 165.28 μg /m^3^ with CO 443 t/year, NO_x_ 876 t/year. By systematical analysis, NO_x_ accounts for 20.5% and 55.5% during take-off and climb phase while CO account for 91.6% during the taxi phase (Yang et al. 2018). In Beirut airport, emission of NO_x_ 454.8 tons (50.7 tons NO_2_, NO 404.1 tons) was also considered as the largest source of main engines and APUs (Mokalled et al. 2018). NO_x_ emission is mainly concentrated in take-off and climb phases accounting for 68.0% of the total emissions while CO emissions were mainly concentrated in the taxi and ground idle phases accounting for 88.0% of the total emissions. CO, and NO_x_ were accounted for 27.5% and 25.7% of the corresponding pollutant emissions in total taxi phase, respectively (Cao et al. 2019). At London Heathrow, NO_x_ emission was found the significant differences by the statistical analysis from the same engine type used on the same aircraft frame (Carslaw et al. 2008). However, It was investigated that fuel chemistry had a significant effect on flame radiation and liner wall temperature, but only a slight effect on the emission of carbon monoxide (CO) and oxide of nitrogen (NO_x_), which were sensibly independent of physical properties over the range of fuels studied (Liu and Yang 2023b; LEFEBVRE and H. A. 1984).

FT-SPK blend appear to show a lower specific fuel consumption (SFC) (Davison, et al. 2015), and several studies confirmed that SAF has a potential energy saving due to a higher heat value (Habib et al. 2010). The Bio-SPK fuel blends showed the improvement in SFC and fuel flow when compared to the Jet A. Both 25% and 50% Bio-SPK blends showed the reduction in fuel flow by 0.7% and 1.2%, respectively, consistent with the improvement of combustion efficiency of 0.6% and 1.1% (Rahmes, et al. 2009).

In comparison with conventional jet fuels, paraffin type of SAFs provides an alternative energy source with PM reduction and UHC reduction. However, various studies related with engine emission in NO_x_ and CO levels were conflicting by different engines (Masiol and Harrison 2014). The reason may be induced by the different level of ambient air quality and the type of engine. The potential effects of UHC and PM in ambient air on engine performance are unclear and deserve investigation.

Previous research mainly focused on the total emissions in airport and subsequently lead to the growth of pollutants in air. However, the total emission as the feedback of descend of air quality could influence engine performance beside engine emission. Therefore, it is beneficial to identify and assess the reciprocal effect of air quality on engine performance and emission. By ZF850 jet engine, the effects of air quality and SAF blend were investigated at the engine level. The critical parameters in fuel composition and ambient air quality were extracted by sensitive analysis. The implication of SAF blend on air quality improvement was assessed. The energy saving and pollutant reduction obtained could be both benefit for air quality improvement in airport and further reduce engine emission as the feedback of less pollutants in ambient air.

## Materials and methods

### Fuel compositions and properties

The compositions of FT, HCHJ and RP-3 fuels were investigated at molecular level by GC–MS (Agilent 7890A/5975C). The chemical substances were captured and identified by using NIST 17(Huang et al. 2022). Carbon distributions, classification distributions, C/H ratios, aromatic concentrations were analyzed based on detailed composition analysis as our previous research (Yang et al. 2016). Classification distributions were classified into n-paraffins, iso-paraffins, cycloparaffins, alkylbenzenes, polycyclic aromatic hydrocarbon (PAH) with above 2 rings according to physicochemical properties.

The density was measured by SYA-1884A (ASTM D4052, ± 1%) while the kinematic viscosity was measured by SYD-265H (ASTM D445, ± 2%). The surface tension was investigated by SFT-A1 (ASTM D1331, ± 0.3%) and net heat value was investigated by HWR-15E (ASTM D5865, ± 1%). In spray test, cone angle and liquid length was investigated by shadow method while Phase SMD and velocity of droplets was investigated by Doppler Anemometry as our previous research (Liu, et al. 2022, 2023b).

### Engine performance and emission indices (EIs)

As the effects of environment and SAF blend on engine emission are complex and interactive impact. The engine tests were designed to assess the impact degree on engine emission by change of air quality, which should create the different air quality environment by engine emission. Air quality conditions were modified by pre-running engine emission. Meanwhile, ambient condition of air quality was detected including PM_2.5_, UHC, CO, NO_x_, CO_2_ and O_2_. The modified air qualities were given in Table 1.

**Table 1:** Air quality condition setting

Condition serial number (engine test mark)	PM_2.5_, mg/m^3^	UHC, ppm	CO, ppm	NO/NO_2_, ppm	CO_2_, %	O_2_, %
Condition 1 (RP-3)	0.0045	0	0	0/0	0.02	21.0
Condition 2 (RP-3^)	0.0355	43	0	0/0.1	0.03	21.13
Condition 3 (RP-3*)	1.01	196	5	0/0.7	0.01	20.99
Condition 4 (RP-3**)	1.45	357	1	0/1	0.01	21.12

Engine emission was described by emission index of PM_2.5_ (g/kg fuel), CO (g/kg fuel), UHC (g/kg fuel), NO_x_ (g/kg fuel) at various thrust settings expressed as mass of substance per unit mass of fuel flow. Carbon dioxide (CO_2_, ± 0.01%) and unburned hydrocarbons (CH_4_, ± 1 ppm) were measured by a nondispersive infrared sensor, while carbon monoxide (CO, ± 1 ppm), nitrogen oxides (NO_x_, ± 0.1 ppm), and sulfur dioxide (SO_2_, ± 1 ppm) were measured by electrochemical sensors. Measurements were conducted once per second. PM_2.5_ was investigated by laser particulate matter analysis instrument (LC-5C, ± 0.001mg/m^3^), given in Table 2.

**Table 2:** Measurement accuracy and error analysis

	Accuracy	Error analysis
Thrust, daN	± 0.03%	< 0.03%
Air flow, kg/s	± 0.1%F·S	1%
Fuel flow, L/min	± 0.5%	< 0.5%
Temperature, ℃	± 1℃	< 0.1%
Speed, r/min	± 0.1%	< 0.1%
CO_2_, %	± 0.01%	< 0.01%
UHC, ppm	± 1 ppm	
CO, ppm	± 1 ppm	
NO, ppm	± 1 ppm	
NO_2_, ppm	± 0.1 ppm	
SO_2_, ppm	± 1 ppm	
PM_2.5_, mg/m^3^	0.001 mg/m^3^	

Carbon species in engine emissions, including UHC, CO, and PM_2.5_, are formed by incomplete combustion and subsequently lead to the loss of heat release. As combustion efficiency can be considered as the important factor related with the combustor performance and fuel consumption, calculated as follows (Liu et al. 2023a):

$$\text{Combustion efficiency }(\text{\%})= \frac{1-({\text{EI}}_{\text{UHC}}\times {\text{HV}}_{\text{UHC}}+{\text{EI}}_{\text{CO}}\times {\text{HV}}_{\text{CO}}+{\text{EI}}_{\text{PM}}\times {\text{HV}}_{\text{PM}}) }{{\text{HV}}_{\text{fuel}}}$$

The ZF850 jet engine was controlled and operating conditions were monitored by a data acquisition system, which could remotely communicate with the engine control unit to set the desired engine speed (Liu and Yang 2023b). The engine performance parameters, including thrust, exhaust gas temperature (EGT), and thrust-specific fuel consumption (TSFC), were recorded every 0.08 s.

## Results and discussion

### Effect of SAF blend on chemical composition and fuel property

Chemical compositions undoubtedly define fuel property including volatility, fluidity, and combustibility. Jet fuel composition can be characterized as classification distribution and carbon distribution (Liu and Yang 2018). From the view of fuel impact, spray performance and combustibility are the crucial factors to influence emission of engine. Fuel spray characteristic is closely related with density, viscosity, surface tension (Liu, et al. 2022, 2023b) while fuel combustion characteristic is closely related with heat value, density, C/H ratio, and aromatics concentration and spatial structure (Liu and Yang 2023b; Liu et al. 2023a).

**Fig. 1:**
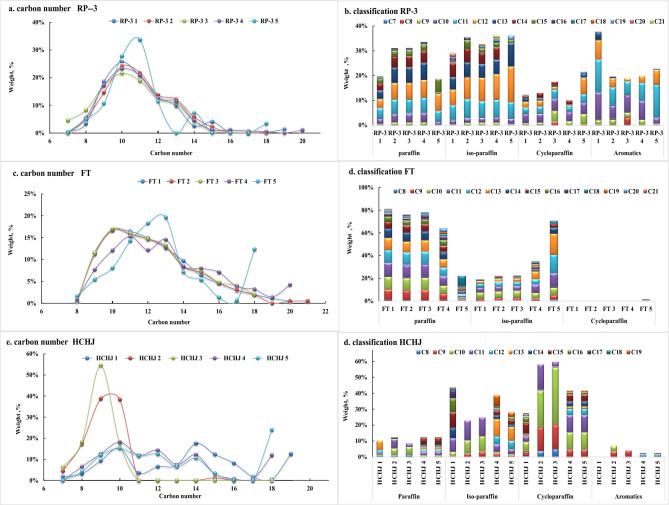
Carbon number and classification of RP-3, FT, HCHJ. **a**. carbon distribution of RP-3; **b**. classification of RP-3; **c**. carbon distribution of FT; **d**. classification of FT; **e**. carbon distribution of HCHJ; **f**. classification of HCHJ

In the respect of composition, the total carbon number distributions of all fuel samples mainly display in the range of C7 to C20 in compliance with distillation requirement of jet fuel. RP-3 fuels as traditional petroleum derived jet fuel present similar normal distributions from C8 to C16 centered on C10 and C11. Iso-paraffins are in a larger range of C8—C20. Cycloparaffins present a normal distribution in the range of C8—C14 while aromatics present a normal distribution in the range of C7—C12, given in Fig. 1a, b. For complying with distillation requirement of drop-in fuel (Liu et al. 2016), FT fuels present a similar normal distribution in the range of C8 to C21, and contain mainly only paraffin and iso-paraffin. Compared with several types of FT fuels, the obvious difference is the ratio of paraffin to iso-paraffin, which could be ascribed to the refining control in isomerization process, given in Fig. 1c, d. Carbon number distributions of HCHJ fuels present in the range of C7-C19 but present obvious distinction of carbon distribution and classification distribution compared with FT. HCHJ fuels (Liu and Yang 2023b; Liu et al. 2023a) are characterized with high cycloparaffin content while FT fuels mainly contain n-paraffin and iso-paraffin. In comparison with HCHJ fuels and FT fuels, RP-3 fuels are characterized with high alkylbenzenes content.

**Fig. 2:**
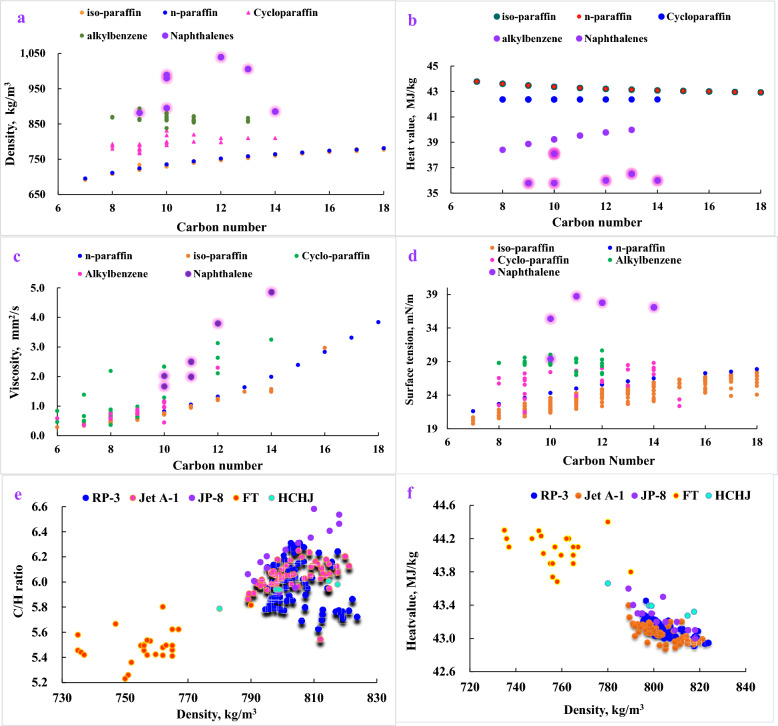
Effects of carbon distribution and classification on fuel properties. **a**. density; **b**. heat value; **c**. viscosity; **d**. surface tension **e**. relationship of C/H ratio with density; **f**. relationship of heat value with density

For sensitive properties on combustion emission, density and heat value both correlates strongly with the enthalpy of combustion per unit mass of fuel while spray performance, influenced by density, viscosity, surface tension, correlates strongly with combustion efficiency and emission. Therefore, the effects of carbon number and classification on key properties were investigated, given in Fig. 2.

Density and heat value are both closely with carbon content in jet fuel. Density increases with carbon content in fuel. The densities of all hydrocarbons increase moderately with the rise of carbon number at the same classification. At the same carbon number, density follows the order as naphthalene > dicycloparaffin > alkylbenzene > cycloparaffin > n-paraffin ~ iso-paraffin, shown in Fig. 2a. Heat value by weight presents the opposite order of density at the same carbon number, and different classification of hydrocarbon appear distinguishable tendency with carbon number. Heat values of aromatic and cycloparaffin grow with carbon number while paraffin and iso-paraffin drop slightly, shown in Fig. 2b.

Viscosity reflects the friction force among molecules in fluid, which is related with molecular size scale and complicated structure. At the same carbon number, naphthalene has slightly higher viscosity than paraffin and alkylbenzene. The viscosity follows the order as naphthalene ~ cycloparaffin > alkylbenzene > n-paraffin > iso-paraffin, shown in Fig. 2c. The paraffin molecules without side chain have greater viscosity than with side chain. The viscosities of n-paraffins increase dramatically with the rise of carbon number while those of iso-paraffins keep almost at the same level.

Surface tension is related with the interaction force among molecules and follows the order as same as density, naphthalene > alkylbenzene > cycloparaffin > n-paraffin > iso-paraffin at the same carbon number. The surface tensions of all hydrocarbons ascend markedly with carbon number, shown in Fig. 2d.

As RP-3, HCHJ, and FT present obviously different in carbon number distribution and classification distribution, properties of blend fuels were detected slight deviation with RP-3 in compliance with drop-in fuel requirement, shown in Fig. 2e, f. From the view of C/H ratio, FT fuels are feature of lower C/H ratio and lower density due to free of aromatics and cycloparaffins while HCHJ fuels have similar C/H ratio as RP-3. In addition, FT fuels have higher heat values than HCHJ fuels and RP-3. Integrating of C/H ratio and heat value, CO_2_ emissions are 70.7 CO_2_ g/MJ FT fuels, 72.3 HCHJ fuels, and 73.2 CO_2_ g/MJ RP-3, respectively.

HCHJ fuels and FTs fuel present slightly higher viscosities and similar surface tensions than petroleum-derived jet fuel of RP-3. Integrating properties effects, cone angles of all blend fuels deviation to RP-3 are below 5%. For liquid length compared with RP-3, FT blends were investigated significant reduction while HCHJ blends were investigated growth, and all deviation were within 5%. For Sauter Mean Diameter (SMD), FT blends were investigated growth around 9% while HCHJ blends were investigated reduction around 5% (Liu et al. 2023b).

### Effects of air quality on engine performance and emission characteristic

**Fig. 3:**
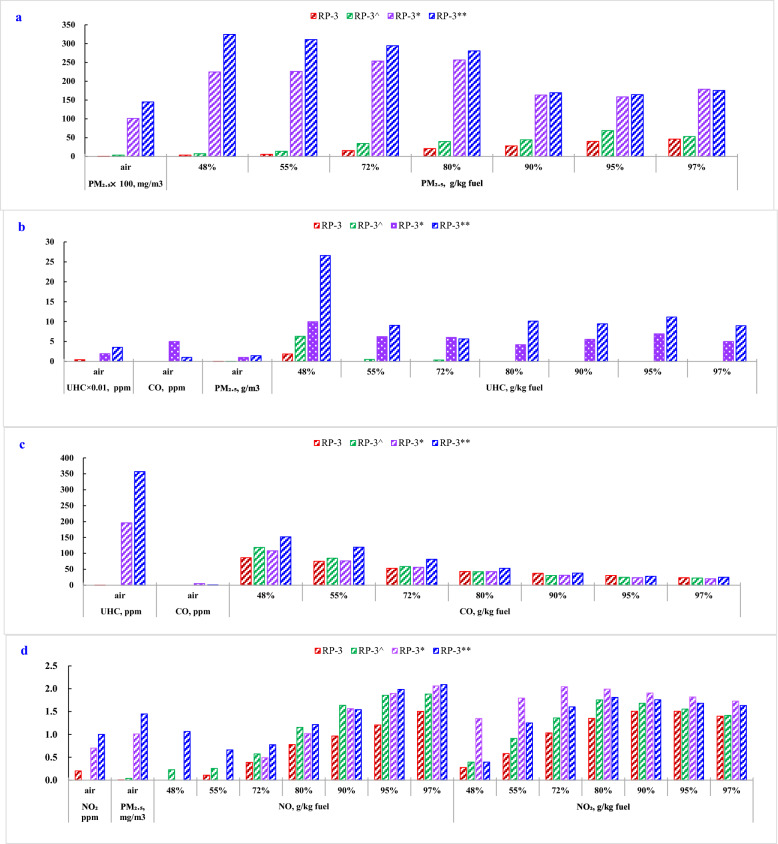
Air quality effects on engine emission at various thrust outputs **a**. PM_2.5_, **b**. UHC, **c**. CO, **d**. NO & NO_x_,

In the respect of emission characteristic, according to the test results, PM_2.5_ emission derived from combustor is not only related with total aromatics in fuel but also PM_2.5_ and UHC in air inlet, both of which could contribute to the rise of PM_2.5_ formation. From Fig. 3a, the same fuel by same engine produces more PM_2.5_ emission at higher PM_2.5_ environment condition than at lower PM_2.5_ environment. PM_2.5_ concentration in air enhanced obviously PM_2.5_ emission of engine.

PM_2.5_ involved in engine by the air inlet could take as the kernel to further enlarge the scale of particle_._ At condition 1 and condition 2 with the lower PM_2.5_ concentration, the largest PM_2.5_ emissions were observed at the higher thrust output setting. However, at condition 3 and condition 4 with higher PM_2.5_ concentration, the largest PM_2.5_ emissions were observed at the lowest thrust output setting. These results indicate that PM_2.5_ emissions should be influenced by engine design and control regulations as well as PM_2.5_ concentration in air. The effects of air quality on PM_2.5_ emission present the reduction with growth of thrust output. but engine emissions at various thrust outputs still are closely related with PM_2.5_ concentration in ambient air.

UHC derived from engine emission can be derived from unburned fuel cracking products, and incomplete combustion products. At low engine thrust output, UHCs are composed of above 90% with double or triple-bonded straight chain hydrocarbons including ethene, ethyne, and propene, therein ethene nearly 50% (Anderson et al. 1994). At higher engine thrust output, aromatic compounds are the dominant compounds (Anderson et al. 1994). From Fig. 3b, UHC emissions derived from engine can be found discernible differences at different air environment. At low engine thrust output (48%), the temperature and pressure of combustor are lower and combustor is less efficient. The UHC emissions all get to the peak at various air environments.

At condition 1 and 2 with less UHC and PM in air, the emissions of many hydrocarbon species dropped at higher engine thrust output and unburned fuel components could disappear, given in Fig. 3b. The results were coincident with the test results by F101 and F110 engines that UHC disappeared at higher engine power (Spicer et al. 1992). At condition 1, UHC disappeared at 55% while UHC disappeared at 80% at condition 2. For condition 3 and 4 with higher UHC and PM in ambient air, UHC decreased by a factor of 2—3 at 97% than at 48%, but UHC as unburned fuel components still can be captured through the whole thrust outputs. At an air environment condition with higher PM_2.5_ and UHC, engine emissions of UHC and PM_2.5_ were enhanced through the whole thrust settings and present wave type.

The effect of air quality on engine emission presents the significant change on PM and UHC. Engine emission increased with the concentration of UHC and PM_2.5_ in ambient air. In additional, the effect degrees on the emission of UHC and PM_2.5_ appear different through the whole thrust settings. Engine enhances thrust output by increasing fuel/air ratio (FAR), which accordingly leads to the growth of flame temperature and shortens the timescale of combustion completeness. The combustor emission depends on combustor operation condition and fuel property as well as combustor structure. Combustor operation condition include inlet temperature and inlet pressure, as well as quality and quantity of air into combustor chamber. Therefore, the quality of air enhanced the engine emission at low engine power due to lower flame temperature and relative longer timescale requirement for combustion completeness. As most of UHC in air can be further oxidized into CO_2_, it is reasonable to deduce the PM could be the main influence factor based on Fig. 3a, b.

In comparison with UHC and PM_2.5_, the effects of air quality on CO and NO_x_ emission present slight changes with a complicated tendency. The opponent role of CO and NO_x_ still present at various thrust settings as previous report (Liu and Yang 2023b). CO emissions are found to be much higher at low engine power, which can be ascribed to lower burning rates and flame temperatures, given in Fig. 3c. Since CO emissions increase with decreasing thrust, these studies confirm that CO emission mainly emit at normal idle and taxi operations in airport (Schafer et al. 2003). However, according to the test results, effect of CO concentration on engine emission shows less difference at different environment condition, which can be deduced that CO in air could react with O_2_ into CO_2_ through combustor. Engine emissions of NO_x_ also show less differences induced by air quality conditions as CO but NO and NO_2_ show both detected differences, given in Fig. 3d.

**Fig. 4:**
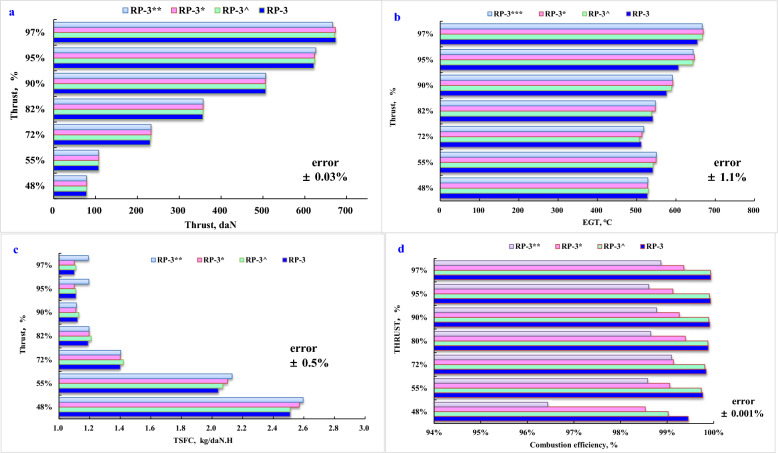
Air quality effects on engine performance at various thrust settings. **a** Thrust, **b**. EGT, **c**. TSFC, **d**. Combustion efficiency

In the respect of engine performance, the test results indicate that there are almost 5% positive benefit in the thrust, EGT, and TSFC through the whole thrust settings compared with air quality at highest PM_2.5_ environment condition, given in Fig. 4a, b, c. Engine performance at various thrust settings still are closely related with PM_2.5_ concentration in ambient air as engine emission.

In general, different environment conditions had a small impact on thrust output as the engine could modify the fuel/air ratio to control the stable thrust output. The effect of air quality on thrust output and EGT present less change, given in Fig. 3a, b. However, the effect of air quality on TSFC and combustion efficiency appear distinguished difference, given in Fig. 3c, d. There is a discernable reduction in TSFC at condition 1 with less emission of PM and UHC. The influence of PM in air is derived from an air inlet of engine. PM could primarily induce fluid friction within compressor blades and accordingly reduce engine efficiency. PM in air may be oxidized in CO_2_ and meantime conduct as a particle kernel to deposit soot, which could also increase fluid friction within the turbine blades (Bester and Yates 2009) and further reduce the overall engine efficiency. For compensating engine efficiency, fuel flow was increased at pollutant environment condition for compliance with the requirement of thrust output. There are almost 3.2% positive benefits in TSFC through the whole thrust settings in ambient air with less pollutants.

In comparison, significant positive combustion efficiencies can be obtained at less pollutant environment conditions. At 48% thrust output, combustion efficiency was 96.4% at condition 4 while combustion efficiency was 99.02% at condition 1. The gap of engine performance among various environment conditions reduced with the rise of thrust output. However, the gap still can be distinguished at even 97% thrust output. At ambient air with higher PM_2.5_ and UHC, combustion efficiencies are 99.05% at condition 3 and 98.87% at condition 4 while combustion efficiencies are 99.93% at condition 1 and 99.89% at condition 2.

### Implications of SAF blend effect on airport

#### Effect of SAF blend on energy saving

**Fig. 5:**
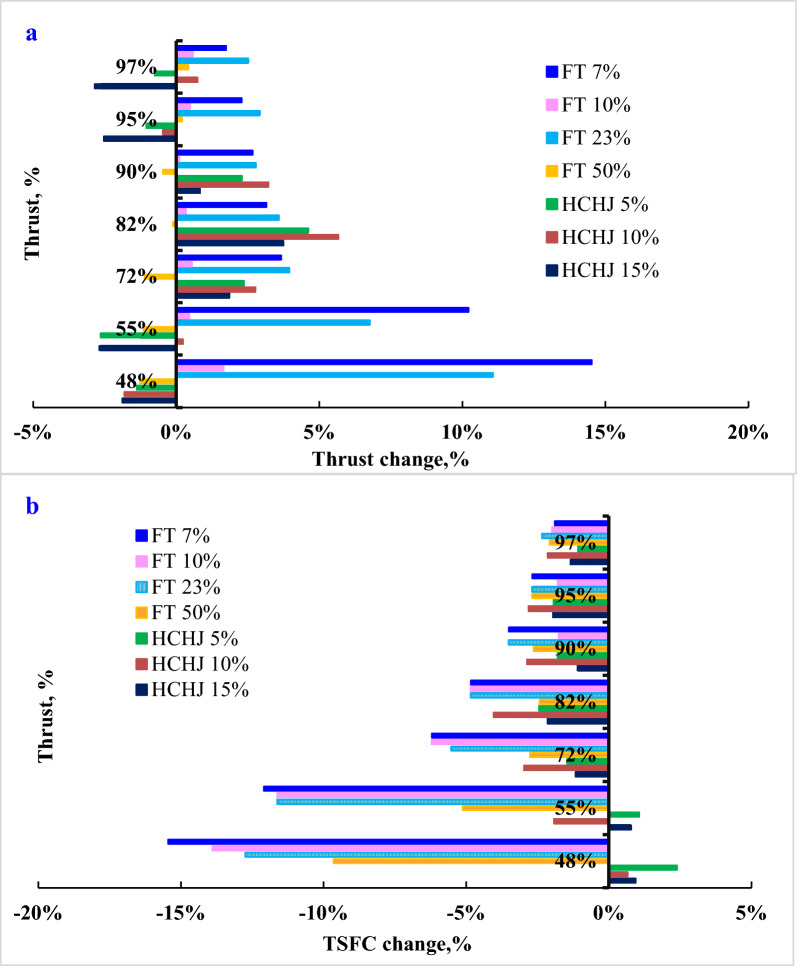
Effects of SAF blend on engine performance at various thrust settings

SAF blends at the various thrust settings are identified for assessing the effects on energy saving in compliance with at same thrust output with RP-3 as the baseline. A series of stable thrust output points of SAF blends were compared with RP-3, given in Fig. 5a. At the various thrust settings, 7%, 10% and 23% FT blend present thrust benefit though the various thrust settings. 14.5% thrust benefit was captured at 48% thrust setting by 7% FT blend fuel compared with RP-3. 5% and 10% HCHJ fuel blends exhibited no obvious differences compared with RP-3, but 15% blend displayed a slight thrust loss at some thrust settings. Both FT blend and HCHJ blend indicate that there exists a range of appropriate blend ratios for obtaining energy saving.

All FT blend fuel conducted consistently lower TSFC than RP-3 in the range of 1.38%—15.4% and are almost 6.67% positive benefit in the TSFC through by 7% FT blend, given in Fig. 5b. Through the whole thrust setting, 10% HCHJ blend fuels conducted the highest reduction of TSFC in HCHJ blends while 7% FT blend fuels conducted the highest reduction of TSFC in FT blends. 5%, 10% and 15% HCHJ blend fuels even enhance slightly TSFC at 48% thrust setting with below 2.39%, which indicated that the different types of SAF present different effect on energy saving. FT fuel composed of mostly paraffins could obtain energy saving in a wide blend range at the whole thrust setting. HCHJ composed of mostly cycloalkanes with some paraffins could obtain energy saving only at the high engine power. The similar investigation has been reported that SAFs composed of mostly cycloalkanes with some aromatics could obtain only a 0.05% savings at the high thrust output condition and had not yet surpassed conventional petroleum fuel at engine-level energy savings at the low power condition (Behnke et al. 2022)

According to a thermodynamic cycle in the gas turbine, higher heat value would increase flame temperature and subsequently a rise of overall thermal efficiency. For keeping the same turbine speed at certain thrust setting, fuel flow of SAF blend could be controlled for keeping the same flame temperature as RP-3. FT blend fuel present the lower C/H ratio with higher heat value, and thus TSFC reductions were obtained at various blends. However, HCHJ blend fuel presents the lower C/H ratio but with slightly higher heat value, and TSFC reductions were obtained slightly only at higher thrust settings. FT fuel with free of aromatics and sulphur lead to high combustion efficiency and subsequently get a benefit in TSFC reduction with PM_2.5_ reduction, which would reduce fluid frictional loss within the turbines and further improved overall engine efficiency. There are almost 5% positive benefits in TSFC through the whole thrust settings by 7% FT blend.

#### Effect of SAF blend on emission reduction

**Fig. 6:**
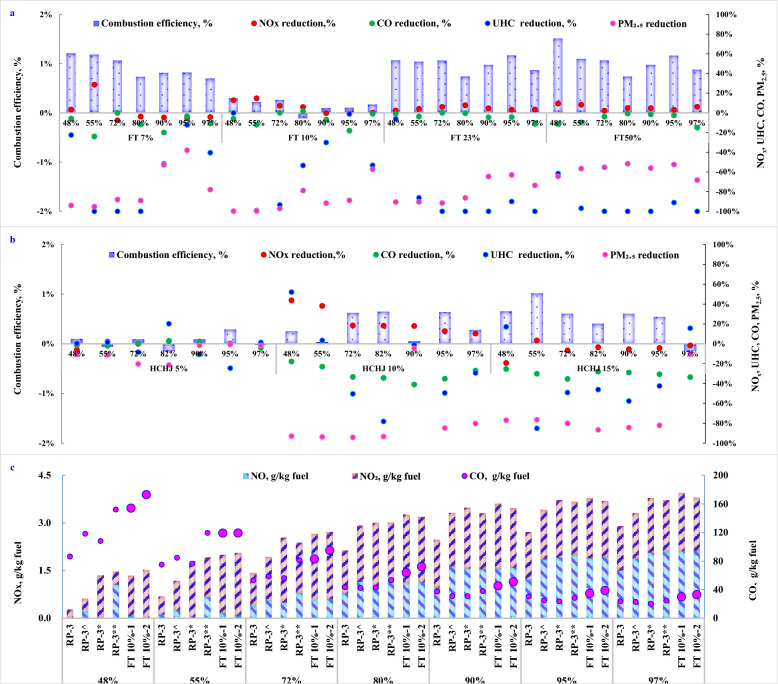
Effects of SAF blend on engine emission

As all aromatic hydrocarbons are easy to form PM_2.5_ and follow the order as naphthalene > cyclo-aromatics > cycloparaffins (Chan et al. 2017), and aromatic content in fuel could increase CO emission (Blakey et al. 2011; Klingshirn, et al. 2012) and UHC emission (DeWitt et al. 2008), FT blend fuels with less aromatics and cycloparaffins and HCHJ blend fuels with less aromatics are both observed significant reductions in PM_2.5_ and UHC, given in Fig. 6a, b.

Engine emission was controlled in compliance with combustor performance. Moreover, PM_2.5_, CO, and UHC are all closely related with thrust setting. C/H ratio defines the enthalpy of combustion per unit mass of fuel and the ratio of H_2_O to CO_2_. In addition, classification distribution and carbon distribution determine radical species (∙H, ∙OH, ∙HO_2_, ∙CH_3_) concentration and distribution, which control the combustion process and emission. Aromatic concentration and oxygen concentration determined the tendency to carbon-containing deposits. Both of blend fuels lead to PM_2.5_ and UHC reduction. PM_2.5_ reduction for FT blend fuels can obtain in the range of 37.9%—99.8% while those of HCHJ blend fuels can obtain in the range of 0.64%-93.9%. All blend fuels present better PM_2.5_ reduction at lower thrust output. UHC reduction perform better at the moderate thrust setting for all blend fuels.

The contrast relationship of CO and NO_x_ appear slight changes at various blend ratios. As nitrogen oxides are produced in the high temperature regions of the combustor primarily through the thermal oxidation of atmospheric N_2_, NO_x_ emissions increase monotonically with engine thrust and is sensitive to temperature profile and concentration of CO. From the view of fuel effects, NO_x_ emissions are still closely related with CO emissions at stable thrust outputs, which conform to a previous research report (Blakey et al. 2011). However, from the view of air quality impact, the relative abundance of NO and NO_2_ are subject to large uncertainties in the plumes. NO in the exhaust can be captured to convert into NO_2_ by the contribution of other emission products (Wood et al. 2008). SAFs blend show less effects on emissions of CO and NO_x_. However, according to Fig. 6c, the effects of air quality on the emissions of CO and NO_x_ present obvious changes at low thrust setting but slight changes at high thrust setting despite of RP-3 or FT. Compared with FT 10%-1(without PM_2.5_) with FT 10%-2 (0.6985 PM_2.5_mg/m^3^), PM_2.5_ could enhance obviously emissions of CO and NO_x_ at 48% thrust setting. The similar results was found that NO_x_ concentrations can differ even using the same airframe and engine type (Carslaw et al. 2008), and the ratio of NO_2_/NO_x_ may vary (Timko, et al. 2010). It is reasonable to deduce that air quality could influence the emissions of CO and NO_x_, specially at the low thrust output.

#### Potential effect of SAF blend on air quality in airport

According to air quality effect on engine performance and emission characteristic, PM and UHC in ambient air are the critical parameters on energy consumption and emission characteristic. Ambient air with PM and UHC is sucked into combustor by air inlet of engine, which can be further combusted into CO_2_ and H_2_O while can be involved as kernel for PM formation. The results confirmed that PM and UHC in air could increase PM and UHC formation in combustor of engine.

The previous research reported that emission characteristics in LTO cycles were detected significant differences carried out by aircraft in different airports (Turgut and Rosen 2010), which may deduce that air quality in airport could lead to significant differences in engine performance and emission characteristic. PM and UHC derived from engine emission are mainly composed of polycyclic aromatic hydrocarbon (PAHs). According to the results from UH-1H engine, total PAHs emissions were made up 59.7% naphthalene, 37.8% three-rings and remaining 2.5% of PAHs with 4—7 rings (Chen et al. 2006). A greater amount of PAH mass was in the vapor phase than in the particle phase. Naphthalene comprised 80%—85% of the total vapor‐phase PAH mass while the semi‐volatile PAHs include phenanthrene and chrysene and the high molecular weight PAHs contain benzo[a] pyrene and indeno [1,2,3‐cd] pyrene (Zhu et al. 2011). PM emitted from aircraft engines by different aircraft engines were dominated by higher molecular weights (> 4 rings) with sulphur-containing substance in 54%—80% and metal elemental emissions within 2%—7% (Kinsey et al. 2011). UHC were dominated by lighter PAHs with 2–3 aromatic rings. Similarity, naphthalene PAHs and its 1-methyl and 2-methyl derivatives are the overwhelmingly dominant PAH species in various aircraft emissions at differing thrust modes (Agrawal et al. 2008). Environmental monitoring in airport was found to be suffering the higher levels of PAHs (27.7 mg/m^3^) with a prevalence of 2—3 ring PAH such as methylnaphthalenes and acenaphthene associated with jet fuel combustion (Cavallo et al. 2006). PM showed a fourfold increase from background concentration levels In Los Angeles international airport (Hudda et al. 2014) and were investigated two orders of magnitude higher at downwind location than upwind locations (Westerdahl et al. 2008). The influence of the naphthalene content to the PAH and soot precursor chemistry could be beyond established correlations such as the hydrogen content (Pelucchi et al. 2021).

SAF blend with feature of lower content of sulfur and aromatic results in reduction of PM and UHC. Based on the average through the whole thrust setting, FT blends could decrease 72.8% PM_2.5_ while HCHJ blends could obtain 52.7% PM_2.5_ reduction. Meanwhile, FT blends could obtain 71.1% UHC reduction while HCHJ blends could obtain 19.9% UHC reduction. The PM_2.5_ mass emissions for the RP-3 and SAF blends fuels correlated well with the variations in the aromatic, hydrogen contents, and H/C ratio in the fuels, and the similar results have been reported (Chan, et al. 2015). For comparison, SAFs result in a tenfold decrease in PM (Moore et al. 2015), and a 5–10% reduction in NO_x_ and CO emissions (Lobo et al. 2012).

Integrating engine performance and emission characteristic, the overall engine benefit was non-linearly related to the blend ratio of SAF blend fuel for both of FT and HCHJ. The results indicated that there is a range of appropriate blend ratio for obtaining the benefit in energy sabing and emission reduction. From the view of energy saving, there are obvious difference between FT blend fuels and HCHJ blend fuels. SAF blend could significantly reduce emission of PM and UHC into air specially at low load thrust in airport, which could decrease engine emission of PM and UHC as feedback, and accordingly decrease the concentrations of PM and UHC in airport.

## Conclusion

UHC and PM_2.5_ concentration in ambient air presents dramatical effects on engine emission in PM_2.5_ and UHC. RP-3 can get 3.2% positive benefits in TSFC through the whole thrust outputs in ambient air with less pollutants. Moreover, combustion efficiency increased 2.6% at low thrust output and 1% at high thrust output.

SAF blend could significantly reduce the concentrations of PM and UHC in airport. FT blends could decrease 72.8% PM_2.5_ while HCHJ blends could obtain 52.7% PM_2.5_ reduction. Meanwhile, FT blends could obtain 71.1% UHC reduction while HCHJ blends could obtain 19.9% UHC reduction. Both FT blend and HCHJ blend indicate that there exists a range of appropriate blend ratios for obtaining benefit of thrust output and energy saving. All FT blend fuel conducted consistently lower TSFC than RP-3 in the range of 1.38%—15.4% but HCHJ can obtain energy saving only at the high engine power, which is consistent with fuel property.

## Data Availability

The datasets generated or analyzed during this study are available from the corresponding author on reasonable request.

## Abbreviations

SAF: Sustainable aviation fuel
EGT: Exhaust gas temperature
TSFC: Thrust-specific fuel consumption
GHGs: Greenhouse gases
RP-3: Petroleum derived jet fuel in China
PAH: Polycyclic aromatic hydrocarbon
PM: Particulate matter
LTO: Landing and take-off
FT: Fischer–Tropsch Synthesized Paraffinic Kerosine
HCHJ: Hydrothermal-condensation-hydrotreating jet fuel (HCHJ)
ATJ: Alcohol-To-Jet
UHC: Unburned hydrocarbon
SMD: Sauter mean diameter
FAR: Fuel/air ratio
